# Clinical-Pathological Conference Series from the Medical University of Graz: Case No 162: A 30-year-old woman from Nigeria with fever 3 months postpartum

**DOI:** 10.1007/s00508-016-1164-9

**Published:** 2017-01-24

**Authors:** Elisabeth Fabian, Bruno Schneeweiss, Thomas Valentin, Holger Flick, Ariane Aigelsreiter, Rainer Hofmann-Wellenhof, Lorenzo Cerroni, Anna Maria Goritschan, Hans-Peter Brezinsek, Sabine Zitta, Alexander Rosenkranz, Winfried Graninger, Guenter J. Krejs

**Affiliations:** 1 Division of Gastroenterology and Hepatology, Department of Internal Medicine III, Medical University of Vienna, Vienna, Austria; 2 Department of Internal Medicine, Hospital Kirchdorf on the Krems, Kirchdorf on the Krems, Austria; 3 Section of Infectious Diseases and Tropical Medicine, Department of Internal Medicine, Medical University of Graz, Graz, Austria; 4 Division of Pulmonology, Department of Internal Medicine, Medical University of Graz, Graz, Austria; 5 Department of Pathology, Medical University of Graz, Graz, Austria; 6 Department of Dermatology and Venerology, Medical University of Graz, Graz, Austria; 7 Division of Nephrology, Department of Internal Medicine, Medical University of Graz, Graz, Austria; 8 Division of Rheumatology and Immunology, Department of Internal Medicine, Medical University of Graz, Graz, Austria; 9 Division of Gastroenterology and Hepatology, Department of Internal Medicine, Medical University of Graz, Auenbruggerplatz 15, 8036 Graz, Austria

**Keywords:** Lymph node tuberculosis, Mycobacterium tuberculosis, Polyarteritis nodosa, Tuberculosis drug therapy

## Presentation of case

### Dr. A.M. Goritschan:

The black African patient had immigrated from Lagos, Nigeria 18 months before admission to Austria to join her husband, who had immigrated to Austria 13 years earlier. Her travel history was negative since arrival in Austria; her medical history was positive for arthralgia and a skin rash 2 years earlier, when she was still living in Nigeria. These symptoms were treated with unspecified injections and oral medication for 5 days. The symptoms subsided when she became pregnant with her first child. After an unremarkable pregnancy and 4 months before the current admission she gave birth to a second healthy child. The patient attended the dermatology outpatient clinic 3 weeks before admission because of a skin rash on her back and fever. She was diagnosed with folliculitis and an ointment with a combination of betamethasone and gentamicin was prescribed for topical treatment. The rash appeared on the hands and feet of the breast feeding woman 2 weeks later with small skin lesions that exsiccated after secretion of a milky exudate. The patient reported fever, mainly during the night and some improvement with paracetamol. An enlarged firm lymph node was palpable in the left axilla. Serology for syphilis and human immunodeficiency virus (HIV) was negative, as was direct examination for scabies. The patient’s laboratory tests were notable for hemoglobin 11.7 g/dl (normal: 12.0–15.3 g/dl), hematocrit 34.8% (normal: 35.0–45.0%), alkaline phosphatase 153 U/l (normal: 35–105 U/l), gamma-glutamyl transferase (GGT) 42 U/l (normal: <38 U/l), aspartate-amino transferase (AST) 37 U/l (normal: <30 U/l), alanine aminotransferase (ALT) 49 U/l (normal: <35 U/l) and C‑reactive protein (CRP) 37.3 mg/l (normal: <5.0 mg/l). At the follow-up visit 2 weeks later the patient reported worsening of her condition. The skin rash was unchanged, but she additionally complained of pain in both lower legs, the ankles and her left foot were swollen. She still had nocturnal fever with temperatures up to 39.0 °C and there was now a second palpable, indolent lymph node in the right axilla. After one of the skin lesions on the thighs was biopsied, the patient and her 4‑month-old daughter were admitted to the department of internal medicine for further work-up.

The patient weighed 68 kg and her height was 170 cm with a body mass index (BMI) of 23.5 kg/m^2^, her neck was supple, her blood pressure was 112/73 mm Hg, and the heart rate was 65 per minute and regular. Except for the two palpable, axillary lymph nodes and the follicular skin rash, the patient complained of pain on firm palpation in both lower legs; both ankles and feet were slightly swollen. The rest of the physical examination was unremarkable, as were the electrocardiogram and chest radiograph. Native computed tomography (CT) of the chest revealed an enlarged lymph node (3.0 × 3.6 cm) in the right axilla. Magnetic resonance imaging (MRI) of the mediastinum was unremarkable, but showed several enlarged lymph nodes in the right axilla with a maximum diameter of 3.6 cm. Lymph nodes in the left axilla were up to 8 mm in diameter. Urinalysis and urinary sediment were within normal limits. At this time the patient’s laboratory tests were notable for CRP 63 mg/l (normal: <5 mg/l), erythrocyte sedimentation rate 86 mm (normal: <20 mm), total protein 7.9 g/dl (normal: 6–8 g/dl), alpha-1-globulin 3.7% (normal: 1.5–3.5%), gammaglobulin 24.3% (10–18.5%) and haptoglobin 3.75 g/l (normal: 0.3–2.0 g/l). The histopathological findings for the skin biopsy showed vasculitis suggesting polyarteritis nodosa but there was no evidence of acute generalized exanthemic pustulous dermatitis (AGEP). Immunopathological parameters were negative; these included antinuclear antibodies (ANA), antineutrophil cytoplasmic antibodies against myeloperoxidase (ANCA-MPO) and proteinase 3 (ANCA-Pr3), antibodies against double-stranded DNA, mitochondria, smooth muscle cells and basal membrane, and screening for extractable nuclear antigens (ENA). At this time, treatment with prednisolone (75 mg daily) was commenced. Consequently, nocturnal fever with recent temperatures up to 40.1 °C disappeared and CRP decreased to 1 mg/l. A blood culture obtained during the night when the patient’s temperature was highest was negative. Serological tests for *Borrelia burgdorferi, Bartonella henselae, Yersinia Types 3* and *9, Toxoplasma gondii, Chlamydia trachomatis, Mycoplasma pneumoniae*, Epstein-Barr virus, Coxsackie A and B viruses, cytomegaly virus, enterovirus, human herpes virus, mumps and parvovirus B 19 were all negative or normal. Angiotensin-converting enzyme (ACE) was also normal. Repeated measurements of procalcitonin did not exceed 0.05 ng/ml (normal 0.03–0.05 ng/ml). Fluorescence-activated cell sorting (FACS) did not indicate a clonal cell population or cells with aberrant expression of markers on leukocytes in the peripheral blood.

A diagnostic test was performed.

## Differential diagnosis

### Dr. B. Schneeweiss:

In essence, the patient under discussion suffers from fever 3 months postpartum, has a skin rash first diagnosed as folliculitis, which presents with small skin lesions drying up after secretion of a milky exudate. In a second phase of the disease the skin manifestations spread over the whole body and the patient developed pain on firm palpation in both lower legs; the ankles and feet were swollen. According to the protocol, the rash was not diagnosed as erythema nodosum. Administration of prednisolone markedly improved her symptoms, a response that could point to a rheumatological disease. This was, however, not confirmed by laboratory data, which were only notable for elevated CRP, blood sedimentation rate and gammaglobulin, and revealed discrete anemia and slightly increased transaminases. Several immunological parameters and screening for ENA were negative, as were ACE, blood cultures and serological tests for various infections. The histopathological findings of the skin biopsy revealed vasculitis and polyarteritis nodosa was suspected. Since the skin lesions were described to secrete milky exudate, Sweet’s syndrome should also be considered in this case.

Furthermore, it is of utmost importance to address the patient’s axillary lymphadenopathy. Depending on the size and localization of the enlarged lymph nodes, different diagnoses should be considered. Various sonographic criteria are available to distinguish benign from malignant lymphadenopathy [1, 2]. Usually, lymphadenopathy is classified as benign if lymph nodes have a diameter <1 cm and infectious and systemic diseases can be excluded. Lymph nodes with a diameter >2 cm often suggest malignancy; however, regardless of the size, the underlying cause of a progression in size of lymph nodes should always be investigated.

In summary, the described symptoms and findings strongly suggest the diagnosis of polyarteritis nodosa. These include (1) lymphadenopathy, (2) swollen feet, which occur in 80% of affected patients, and (3) fever, which is observed in 25–30% of cases. Furthermore, the prompt positive response to corticosteroids described in this case is typical for polyarteritis nodosa [3] as are normal immune parameters, such as ANCAs, also observed in this patient [4]. Approximately 50% of patients with polyarteritis nodosa present with livedo reticulatis, which, however, is difficult to diagnose in people with dark skin. On the other hand, the histopathological finding of vasculitis in this case does not strongly suggest polyarteritis nodosa, which is defined as necrotizing arteritis not associated with ANCAs of medium or small arteries without glomerulonephritis or vasculitis in arterioles, capillaries or venules [5]. Additionally, no organ is documented to be involved, which is also atypical for polyarteritis nodosa. Thus, other causes for lymphadenopathy and bullous skin rash in relation to rheumatological and infectious diseases have to be considered (Table 1). Although systemic lupus erythematosus (SLE) may present with negative serology (as observed in the discussed patient), the course of the disease in this case does not suggest SLE. Behcet’s disease can also be excluded because this disease does not occur in black people [6] and with negative radiograph, CT and MRI, there are no indications for a granulomatous disease such as Wegener’s granulomatosis or lymphomatoid granulomatosis. Although serology was negative for various infections, at this point infection with *Mycobacterium tuberculosis* cannot be ruled out. In my experience this infection can present with a wide range of symptoms. Other diseases (Table 1) that might cause lymphadenopathy and skin rash include inflammatory bowel disease (IBD). Both are typical symptoms in IBD and in some cases they present many years before intestinal manifestations of the disease. Cutaneous symptoms in IBD include vulvoperineal Crohn’s disease, genital lymphedema, lymphangioma circumscripta, orofacial granulomatosis, erythema nodosum, pyoderma gangrenosum, peristomal pyoderma gangrenosum, pyostomatitis vegetans, oral aphthous ulcers, Sweet’s syndrome, leukocytoclastic vasculitis, cutaneous polyarteritis nodosa and epidermolysis bullosa acquisita [7]. Several cases of cutaneous polyarteritis nodosa in Crohn’s disease have been described [7–9] and may also be suspected here. It should also be considered that cutaneous polyarteritis nodosa can be induced by *Mycobacterium tuberculosis* [10]. This limits our patient’s diagnosis to IBD, most likely Crohn’s disease, or mycobacterial infection. To finally establish the right diagnosis in this case I would propose tests for tuberculosis including an interferon-gamma release assay (IGRA), imaging studies of the abdomen and biopsy of an axillary lymph node. Since the axillary lymph nodes with a diameter up to approximately 4 cm are highly pathological, I strongly suspect tuberculosis in this patient.

**Table 1:** Causes for lymphadenopathy combined with a skin rash as described in the patient

Papulonodular rash	Vesicular, bullous rash
*Rheumatological diseases*	
Systemic lupus erythematosus Inflammatory bowel disease Behcet’s syndrome Polyarteritis nodosa Wegener’s granulomatosis Lymphomatoid granulomatosis	Systemic lupus erythematosus Inflammatory bowel disease Behcet’s disease Neutrophilic dermatoses Wegener’s granulomatosis Kawasaki disease
*Infections*	
Neisserial infections Subacute bacterial endocarditis Syphilis Fungal infections Mycobacterial infections	Neisserial infections Lyme disease Viral infections

## Dr. B. Schneeweiss’s diagnosis

Tuberculosis.

## Discussion of case

### Dr. A. Aigelsreiter:

**Fig. 1:**
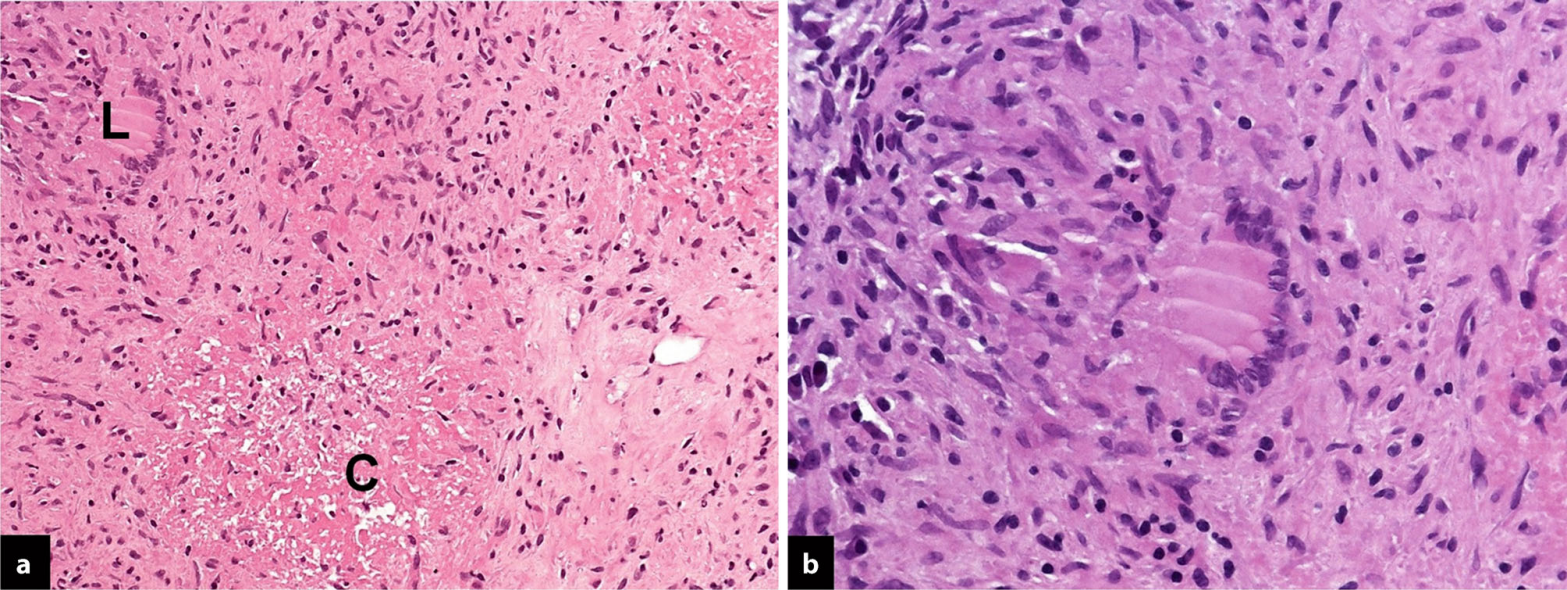
**a** Overview of epithelioid cell granuloma. In the center a necrotizing caseating  area (*C*) surrounded by epithelioid cells and lymphocytes, with a multinucleated Langerhans giant cell (*L*) (H&E, x20). **b** Higher magnification of the multinucleated Langerhans giant cell in the middle surrounded by epithelioid cells and lymphocytes (H&E, x60)

A sonographically guided biopsy of the enlarged right axillary lymph node was performed in the breast center of the department of obstetrics and gynecology. Histopathology revealed destroyed lymph node structure, as well as necrotizing caseating areas surrounded by epithelioid cell granulomas and lymphocytes; multinucleated Langerhans giant cells were also present (Fig. 1). Caseating granulomas are classically associated with mycobacterial infections but can also occur with fungal infection (e. g. histoplasmosis), tularemia and rarely sarcoidosis [11]. For the pathologist, two diagnoses have to be primarily considered in this case with granulomatous lymphadenitis: tuberculosis and sarcoidosis. Staining for acid-fast bacilli was negative. PCR testing for *Mycobacterium tuberculosis* was positive and finally led to the diagnosis of lymph node tuberculosis.

### Dr. G.J. Krejs:

Dr. Flick is a specialist for tuberculosis and will present the course of the disease in this patient.

### Dr. H. Flick:

On firm diagnosis of lymph node tuberculosis, the patient received standard treatment for tuberculosis with quadruple drug therapy (rifampicin 600 mg q.d., isoniazid 300 mg q.d., pyrazinamide 1500 mg q.d., ethambutol 1000 mg q.d.) for 2 months and a 2-drug therapy (rifampicin 600 mg q.d., isoniazid 300 mg q.d.) for an additional 4 months. Prednisolone was tapered but had to be reintroduced (50 mg per day) because her vasculitis had worsened. After 2 months of treatment for tuberculosis, CT showed that the enlarged axillary lymph nodes were now much smaller. The patient became completely free of symptoms and had gained weight under long-term therapy with prednisolone. Currently, she is still under two-drug therapy for tuberculosis and additionally receives corticosteroids, which will be slowly tapered during the next months.

### Dr. G.J. Krejs:

Therapy with corticosteroids in tuberculosis is considered dangerous and is usually avoided because it could trigger spread of the infection.

### Dr. B. Schneeweiss:

To my knowledge, in addition to the tuberculostatic therapy, patients with tuberculous meningitis are also treated with corticosteroids despite the infection with *Mycobacterium tuberculosis*. In this case, however, the remarkably quick and positive response of the patient to corticosteroids is a surprising and interesting observation.

### Dr. R. Hofmann-Wellenhof:

**Fig. 2:**
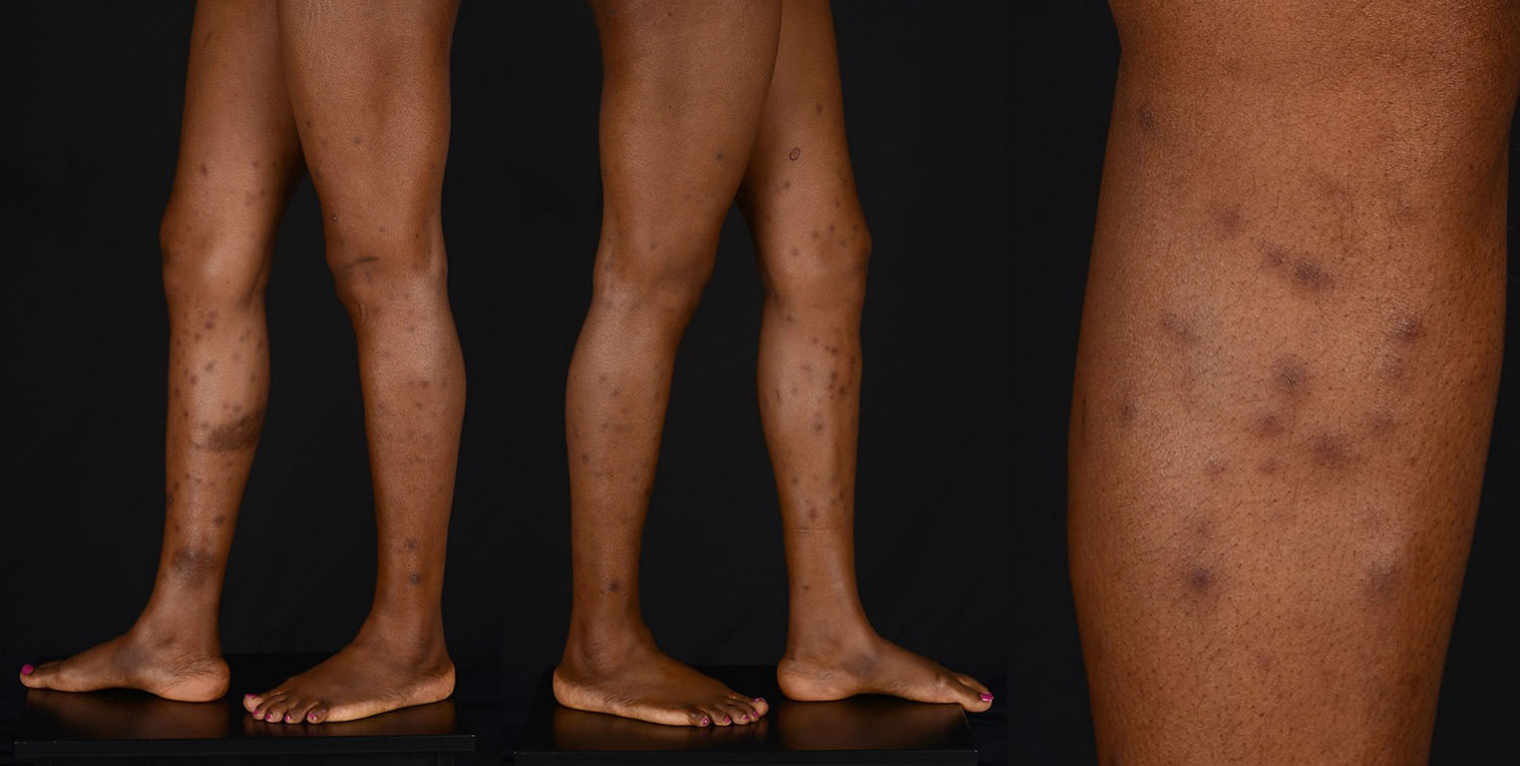
Skin rash on the lower legs with multiple papules up to 1.5 cm in diameter. On the right leg two large hyperpigmented macules

**Fig. 3:**
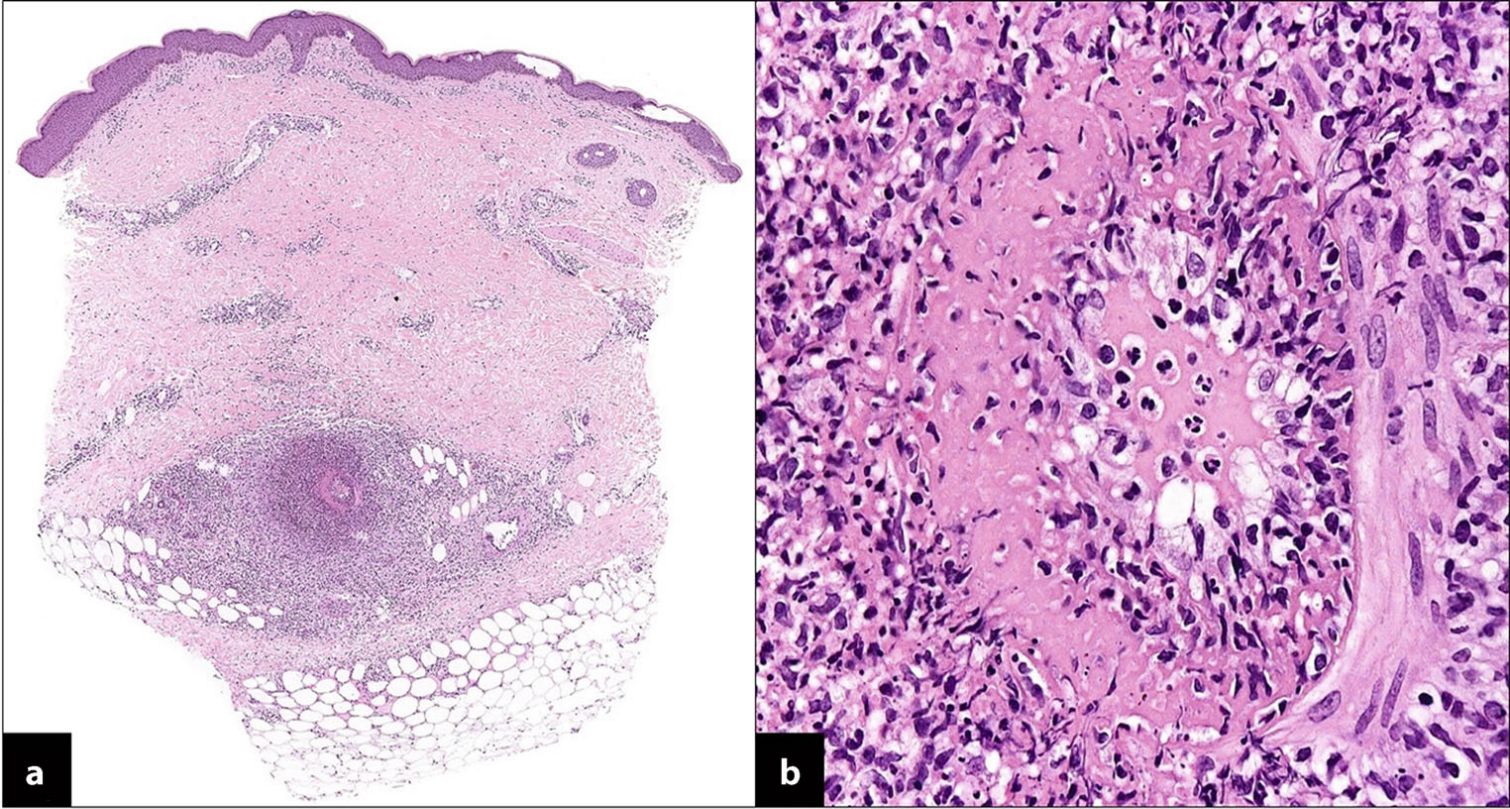
Histology of a skin biopsy revealed **a** vasculitis of a mid-sized vessel located in the superficial subcutaneous fat (H&E, x20); **b** detail showing a dense inflammatory infiltrate centered around and infiltrating a mid-sized vessel (H&E, x100)

When the patient first presented at the dermatology outpatient clinic, her skin condition was diagnosed as folliculitis, i. e. follicular-associated inflammation. Folliculitis is frequently observed in febrile diseases as reported by the patient because of increased perspiration. After 3 weeks of local therapy with an ointment combining steroids and antibiotics, the folliculitis disappeared and the patient presented with a papulous rash (Fig. 2) with lesions too small and superficial for erythema nodosum. Histology of a biopsy taken from a skin lesion on her thighs revealed dense infiltration of the tissue with lymphocytes and macrophages in the cutis and subcutis. Inflammation was mainly seen around medium and small vessels (Fig. 3). Histology of the skin lesion did not reveal granulomatous inflammation or caseating granulomas. Superficial vessels were not affected by inflammation as is typical for superficial allergic vasculitis, which presents with superficial necrotizing skin lesions. Clinical symptoms and histopathology led to the diagnosis of polyarteritis nodosa. This suspicion was further strengthened by the patient’s positive response to corticosteroids. Further tests, however, ultimately led to the diagnosis of tuberculosis. Erythema induratum of Bazin, which is typically but rarely observed in tuberculosis and characterized by large papulous plaques and inflammation also affecting the subcutis was not found and PCR of the skin biopsy for *Mycobacterium tuberculosis *was negative. According to the literature, however, pathogens are rarely detected in skin biopsy [12, 13]. Taking together the skin manifestations and lymph node tuberculosis, the skin lesions have to be interpreted as tuberculosis-associated immunologically mediated vasculitis.

### Dr. G.J. Krejs:

Since the patient complained of swollen and painful ankles, a rheumatologist was consulted.

### Dr. H.-P. Brezinsek:

Diagnosis of a rheumatological disease was quite difficult because the patient had already been under corticosteroid therapy and the ankle pain as well as the painful skin rash had subsided when I examined her. Basically, tuberculosis can also affect joints, especially the spine, hips and knees, but these structures were not affected. In suspected cases, joint puncture and PCR testing for *Mycobacterium tuberculosis* of the punctate should be performed to confirm a joint affection by tuberculosis. Involvement of the ankle joints and even the described course of the joint symptoms in this patient would have been very atypical for tuberculosis, so that it seems very unlikely that the joint problems were indeed due to tuberculosis; they are more compatible with sarcoidosis, e. g. Löfgren’s syndrome, but this syndrome also includes erythema nodosum, which this patient did not have. Because of the rapid response to the treatment, we concluded that the symptoms were parainfectious. Unfortunately, no imaging studies (ultrasound or MRI) of her joints were made before corticosteroids were started. We can only speculate as to whether her symptoms were just arthralgia, commonly seen during infections, or arthritis. Reactive arthritis would be unlikely since the main criteria for diagnosis [14], e. g. enteritis and urethritis, were not present.

### Dr. G.J. Krejs:

Despite advances in diagnosis and therapy, tuberculosis is still one of the world’s biggest health threats and a major public health concern; decades ago the World Health Organization established a global tuberculosis monitoring system [15]. It is of interest that in western countries, while the total incidence of tuberculosis decreased, the proportion of extrapulmonary tuberculosis increased from 8% in 1962 to 21% in 2015 [11, 16–18]. The most common type of extrapulmonary tuberculosis is lymph node tuberculosis followed by pleural, genitourinary, bone/joint, miliary, meningeal and peritoneal manifestation of the disease [19].

Dr. Flick will provide further epidemiological data on tuberculosis, specifically focusing on the situation in Austria.

### Dr. H. Flick:

According to the national reference center for tuberculosis, the total number of cases in Austria decreased from 1480 cases in 1997 to 582 in 2014, for a yearly incidence of 6.8 per 100,000 population. As in other high-income countries, the incidence of tuberculosis is generally higher in foreign-born persons (31.2 per 100,000 population in 2014) than in Austrian-born persons (3.3 per 100,000 population in 2014), with 82% presenting with pulmonary tuberculosis and 18% with extrapulmonary manifestations [20]. The linear decrease of tuberculosis incidence in Austria (‑0.65 per 100,000 population per year from 1997 to 2014) is attributable to a reduction of cases in native-born persons; the incidence in foreign-born persons in Austria tends to decrease only very slightly, and is still more than ten-fold higher than in native-born persons. This discrepancy in the incidence of tuberculosis among native-born and foreign-born persons may be explained by the fact that in Austria transmission of tuberculosis is effectively limited by immediate isolation of infected patients. This is important because each case of active tuberculosis is estimated to cause 10–20 further infections [21]. In contrast, detection of active tuberculosis and isolation of infected patients is not routine practice in many low-income countries with lower health care standards. Depending on the intensity of exposure to *Mycobacterium tuberculosis,* up to 30% of individuals are estimated to develop latent infection after contact with an infectious index patient [22] and one third of the world’s population is estimated to be latently infected with *Mycobacterium tuberculosis* [23]. These individuals carry a 5–10% lifetime risk of progressing to active tuberculosis [24]. Treatment of latent infections with isoniazid reduces the risk of future disease by 75–90% [25]. Undetected tuberculosis (active and latent) is a major problem in low-income countries and flight and migration of people from countries with high tuberculosis incidence may additionally enhance the risk of transmission among immigrants.

If a foreign-born patient (particularly from high incidence regions such as Asia and Africa) presents with fever and unclear symptoms, tuberculosis should be suspected. The discussed patient came from Nigeria, where the yearly incidence of tuberculosis is about 300–499 per 100,000 population [15]. Physicians should be aware of tuberculosis, particularly when clinical symptoms, travel history or migration strongly suggest the disease. When tuberculosis is suspected, a tissue biopsy should be examined with acid-fast bacilli staining, culture, and PCR, as was done in this case.

For the diagnosis of tuberculosis, it should be borne in mind that both the tuberculin skin test (TST) and interferon gamma release assay (IGRA) have highly variable sensitivity, even in active cases of tuberculosis. In patients with culture-confirmed tuberculosis, the sensitivity of the TST ranges from 75–90%; however, cross-reactivity of the intradermally injected purified protein derivate of *Mycobacterium bovis* with nontuberculous mycobacteria and the bacillus Calmette Guérin vaccine limits the specificity of the test in a population [26]. It has further been shown that TST is negative in up to half of all cases of disseminated tuberculosis [11]. The IGRA measures interferon gamma response of the immune system to infection in blood samples using highly specific *Mycobacterium tuberculosis* antigens, thus providing improved specificity over TST [27, 28]. However, IGRA lacks the sensitivity that is routinely demanded from other laboratory tests; false negative test results are reported in up to 25% of cases [27]. Both TST and IGRA do not provide information on whether the disease is active or latent and a negative test does not exclude tuberculosis. False negative results may delay the correct diagnosis and treatment because physicians will probably want to rule out infection with *Mycobacterium tuberculosis* early and then search for other causes of disease. In this patient the IGRA was first negative and later, after the diagnosis was already established, became positive.

As observed here, immunological reactions and vasculitis in various organs are very rare, but well described. Depending on the affected organ, this can cause severe health problems in tuberculosis, especially when the central nervous system (CNS) is involved. To reduce the risk of increased immune reactions leading to vasculitis, concurrent therapy with anti-tuberculous drugs and corticosteroids is recommended in CNS tuberculosis [29, 30]. In other cases of tuberculosis, administration of corticosteroids is, however, controversial because it may worsen the response to tuberculosis therapy due to the modulation of immune reactions.

### Dr. G.J. Krejs:

Dr. Harnoncourt as a pulmonologist will briefly report his experience with tuberculosis.

### Dr. K. Harnoncourt:

This case reminds me of a 65-year-old nun with lymph node tuberculosis whom I treated almost 60 years ago. She presented with an enlarged lymph node and poor general health. Since tuberculosis was suspected, a lymph node biopsy was tested for *Mycobacterium tuberculosis*. Back then this was done in animals, i. e. mice were inoculated with material obtained from the patient. Approximately 6 weeks later this “bioassay” proved *Mycobacterium tuberculosis* infection in our patient. In the 1950s patients received a two-drug antituberculous standard therapy (isoniazid and streptomycin) for 6 months with a reduction in dosage after 4 months.

### Dr. A. Rosenkranz:

As already mentioned, extrapulmonary tuberculosis generally carries little risk for transmission. In this case, even breastfeeding did not transmit the disease to the baby, which was tested after the mother’s diagnosis had been established. Under quadruple antituberculous therapy, however, the baby had to be weaned. Regarding the risk of transmission, I would like to mention that normal serum procalcitonin levels as observed in our patient rather suggest an immunological than an infectious cause of her symptoms [31].

### Dr. G.J. Krejs:

In 2017 tuberculosis remains an important diagnostic consideration. In the 30-year-history of our clinical-pathological conference (CPC) series from the Medical University of Graz, including this case we discussed a total of 5 cases of tuberculosis, or 3% of all CPCs. There were two cases of tuberculous peritonitis, one of tuberculous spondylodiscitis, one of ileocecal tuberculosis, and the current patient, who suffered from lymph node tuberculosis.

### Dr. B. Schneeweiss:

This case clearly highlights that even in 2017 tuberculosis should be considered when a patient presents with fever and unclear symptoms, particularly when coming from a country in which the disease is endemic.

## Final diagnosis

Lymph node tuberculosis, tuberculosis-associated vasculitis mimicking polyarteritis nodosa.

## References

[1] Hines CM, Toy EC, Barker B (2001). The clinical evaluation of lymphadenopathy. Prim Care Update Ob Gyns.

[2] Jung W, Trümper L (2008). Differentialdiagnose und -abklärung von Lymphknotenvergrößerungen. Internist.

[3] Morgan AJ, Schwartz RA (2010). Cutaneous polyarteritis nodosa: a comprehensive review. Int J Dermatol.

[4] Howard T, Ahmad K, Swanson JA, Misra S (2014). Polyarteritis nodosa. Tech Vasc Interv Radiol.

[5] Jennette JC, Falk RJ, Bacon PA, Basu N, Cid MC, Ferrario F, Flores-Suarez LF, Gross WL, Guillevin L, Hagen EC, Hoffman GS, Jayne DR, Kallenberg CG, Lamprecht P, Langford CA, Luqmani RA, Mahr AD, Matteson EL, Merkel PA, Ozen S, Pusey CD, Rasmussen N, Rees AJ, Scott DG, Specks U, Stone JH, Takahashi K, Watts RA (2013). 2012 revised International Chapel Hill Consensus Conference Nomenclature of Vasculitides. Arthritis Rheum.

[6] Ndiaye M, Sow AS, Valiollah A, Diallo M, Diop A, Alaoui RA, Diatta BA, Ly F, Niang SO, Dieng MT, Kane A (2015). Behçet’s disease in black skin. A retrospective study of 50 cases in Dakar. J Dermatol Case Rep.

[7] Hagen JW, Swoger JM, Grandinetti LM (2015). Cutaneous manifestations of Crohn disease. Dermatol Clin.

[8] Gudbjörnsson B, Hällgren R (1990). Cutaneous polyarteritis nodosa associated with Crohn’s disease. Report and review of the literature. J Rheumatol.

[9] Dyer NH, Verbov JL, Dawson AM, Borrie PF, Stansfeld AG (1970). Cutaneous polyarteritis nodosa associated with Crohn’s disease. Lancet.

[10] Imanishi H, Tsuruta D, Oshimo T, Sowa J, Mizuno N, Nakagawa K, Ishii M (2012). Cutaneous polyarteritis nodosa induced by Mycobacterium tuberculosis. J Dermatol.

[11] Abad CL, Moseley RH, Crnich CJ, Saint S, Safdar N (2014). A gut instinct. N Engl J Med.

[12] Tan SH, Tan BH, Goh CL, Tan KC, Tan MF, Ng WC, Tan WC (1999). Detection of Mycobacterium tuberculosis DNA using polymerase chain reaction in cutaneous tuberculosis and tuberculids. Int J Dermatol.

[13] Kim HM, Park YB, Maeng HY, Lee SK (2006). Cutaneous leukocytoclastic vasculitis with cervical tuberculous lymphadenitis: a case report and literature review. Rheumatol Int.

[14] Braun J, Kingsley G, van der Heijde D, Sieper J (2000). On the difficulties of establishing a consensus on the definition of and diagnostic investigations for reactive arthritis. Results and discussion of a questionnaire prepared for the 4th International Workshop on Reactive Arthritis, Berlin, Germany, July 3–6, 1999. J Rheumatol.

[15] WHO (2015). Global Tuberculosis Report 2015. 20th ed, World Health Organization, 2015.

[16] CDC (2007). Reported tuberculosis in the United States, 2006.

[17] Peto HM, Pratt RH, Harrington TA, LoBue PA, Armstrong LR (2009). Epidemiology of extrapulmonary tuberculosis in the United States, 1993–2006. Clin Infect Dis.

[18] CDC (2015). Reported tuberculosis in the United States, 2014.

[19] Mehta JB, Dutt A, Harvill L, Mathews KM (1991). Epidemiology of extrapulmonary tuberculosis. A comparative analysis with pre-AIDS era. Chest.

[20] Österreichische Agentur für Gesundheit und Ernährungssicherheit (AGES) (2014). Annual report of the Austrian National Reference Center for Tuberculosis 2014.

[21] Shaw JB, Wynn-Williams N (1954). Infectivity of pulmonary tuberculosis in relation to sputum status. Am Rev Tuberc.

[22] Grzybowski S, Barnett GD, Styblo K (1975). Contacts of cases of active pulmonary tuberculosis. Bull Int Union Tuberc.

[23] WHO (2015). Guidelines on the management of latent tuberculosis infection.

[24] Comstock GW, Cauthen GM, Reichman L, Herschfield E (1993). Epidemiology of tuberculosis. Tuberculosis. A comprehensive approach.

[25] WHO (2009). Global tuberculosis control: epidemiology, strategy, financing.

[26] American Thoracic Society (2000). Trageted tuberculin testing and treatment of latent tuberculosis infection. MMWR Recomm Rep.

[27] Herrera V, Perry S, Parsonnet J, Banaei N (2011). Clinical application and limitations of interferon-gamma release assays for the diagnosis of latent tuberculosis infection. Clin Infect Dis.

[28] Andersen P, Munk ME, Pollock JM, Doherty TM (2000). Specific immune-based diagnosis of tuberculosis. Lancet.

[29] Thwaites GE, Nguyen DB, Nguyen HD, Hoang TQ, Do TT, Nguyen TC, Nguyen QH, Nguyen TT, Nguyen NH, Nguyen TN, Nguyen NL, Nguyen HD, Vu NT, Cao HH, Tran TH, Pham PM, Nguyen TD, Stepniewska K, White NJ, Tran TH, Farrar JJ (2004). Dexamethasone for the treatment of tuberculous meningitis in adolescents and adults. N Engl J Med.

[30] Thwaites G, Fisher M, Hemingway C, Scott G, Solomon T, Innes J, British Infection Society (2009). British Infection Society guidelines for the diagnosis and treatment of tuberculosis of the central nervous system in adults and children. J Infect.

[31] Ospina FE, Echeverri A, Zambrano D, Suso JP, Martínez-Blanco J, Cañas CA, Tobón GJ (2016). Distinguishing infections vs flares in patients with systemic lupus erythematosus. Rheumatology (Oxford).

